# *Drosophila* Growth Cones Advance by Forward Translocation of the Neuronal Cytoskeletal Meshwork *In Vivo*

**DOI:** 10.1371/journal.pone.0080136

**Published:** 2013-11-11

**Authors:** Douglas H. Roossien, Phillip Lamoureux, David Van Vactor, Kyle E. Miller

**Affiliations:** 1 Cell and Molecular Biology Program, Michigan State University, East Lansing, Michigan, United States of America; 2 Department of Zoology, Michigan State University, East Lansing, Michigan, United States of America; 3 Department of Cell Biology, Harvard Medical School, Boston, Massachusetts, United States of America; Stanford University School of Medicine, United States of America

## Abstract

*In vitro* studies conducted in *Aplysia* and chick sensory neurons indicate that in addition to microtubule assembly, long microtubules in the C-domain of the growth cone move forward as a coherent bundle during axonal elongation. Nonetheless, whether this mode of microtubule translocation contributes to growth cone motility *in vivo* is unknown. To address this question, we turned to the model system *Drosophila*. Using docked mitochondria as fiduciary markers for the translocation of long microtubules, we first examined motion along the axon to test if the pattern of axonal elongation is conserved between *Drosophila* and other species *in vitro*. When *Drosophila* neurons were cultured on *Drosophila* extracellular matrix proteins collected from the *Drosophila* Kc167 cell line, docked mitochondria moved in a pattern indicative of bulk microtubule translocation, similar to that observed in chick sensory neurons grown on laminin. To investigate whether the C-domain is stationary or advances *in vivo*, we tracked the movement of mitochondria during elongation of the aCC motor neuron in stage 16 *Drosophila* embryos. We found docked mitochondria moved forward along the axon shaft and in the growth cone C-domain. This work confirms that the physical mechanism of growth cone advance is similar between *Drosophila* and vertebrate neurons and suggests forward translocation of the microtubule meshwork in the axon underlies the advance of the growth cone C-domain *in vivo*. These results highlight the need for incorporating en masse microtubule translocation, in addition to assembly, into models of axonal elongation.

## Introduction

While there has been immense success in identifying the proteins that control and contribute to axonal elongation [1,2], the mechanical process of growth cone motility has received comparatively little attention. Recent studies now suggest that that in addition to microtubule assembly, growth cone advance is paired with forward translocation of the entire microtubule bundle along the axon and in the growth cone [3–5]. This opens the exciting possibility of developing new models of axonal elongation [6]. Yet because growth is sensitive to the context of the extracellular environment, whether these new findings *in vitro* are relevant to growth cone motility *in vivo* is unknown. 

Growth cones are typically divided into three major structural regions: an actin rich peripheral domain (P-domain) that undergoes retrograde flow, a microtubule and organelle rich central domain (C-domain) that advances at the same rate as axons elongate, and a transition zone (T-zone) between these domains where the plus ends of microtubules interact with actin arcs [2]. The adjoining axon consists of a meshwork of cortical actin filaments and spectrin [7–9] that surrounds a core of cross-linked microtubules [10]. Embedded within this meshwork are organelles that are stably linked to microtubules [11], actin [12], and neurofilaments [13,14] which is beautifully illustrated in classic electron micrographs [8]. While the dynamics of actin in the peripheral domain of the growth cone are relatively well understood in terms of a molecular clutch [15] that links [16] actin retrograde flow with the generation of traction forces [17,18] and protrusion at the leading edge, the movement patterns of microtubules in the C-domain and axon [6] are still poorly understood. 

The prevailing theory of axonal elongation, called the Protrusion, Engorgement, and Consolidation (PEC) hypothesis [2,19] classically proposed that the meshwork of cytoskeletal elements in the C-domain and along the axon is stationary [19,20] and growth cone advance is directly coupled with microtubule assembly in the growth cones [21,22] as well as Kinesin / Dynein based delivery of new cytoskeletal elements and organelles to the tip of the axon [23–26]. The Stretch and Intercalated (SAI) growth hypothesis [6,27], extends this model by proposing that in addition to microtubule polymerization, forces pull and / or push the axonal microtubule mass forward causing the C-domain to move forward relative to the substrate [6]. In the SAI model at a microscopic level, much like the stop-and-go transport hypothesis [24,28], translocation occurs because forces cause microtubules and other cytoskeletal filaments to slide apart [29]. But to be clear there is a dramatic difference between the microtubule translocation that occurs by SAI and Stop-and-Go transport. During Stop-and-Go microtubules move at a rate of approximately 0.1 - 1 μm/sec (i.e. 360 - 3600 µm / h) as short filaments down long microtubules [28]. In SAI, long microtubules move as a cross-linked meshwork at the slow rate of 1 - 50 µm / h in the distal axon [27]. 

While microtubules have been a central focus in the study of axonal elongation, their slow translocation is difficult to track using photoactivation or photobleaching because they are dynamic [30]. While fluorescent speckle microscopy could potentially overcome this limitation [31], because it requires high levels of illumination the resulting photo-damage makes it difficult to routinely image microtubules over extended periods of time. Our approach to this problem has been to use docked mitochondria as a fiduciary marker for the movement of the cytoskeletal meshwork [3,6,32]. Following fast transport by Kinesin-1 and dynein [33], mitochondria ‘dock’ to microtubules [11], actin filaments [12], and in vertebrates directly to neurofilaments [13,14]. Once mitochondria are docked they remain in position for hours. Facilitating the analysis of mitochondria transport, they are easy to label with fluorescent dyes [34] and GFP targeted to mitochondria [33]. Furthermore, they can be monitored using low levels of illumination that minimally impair axonal elongation [27]. The use of mitochondria to track the movement of the cytoskeletal meshwork has been validated in prior studies that have demonstrated that beads bound to the axonal actin cortex, axonal branch points, and docked mitochondria all translocate forward during axonal elongation [27]. For all three this occurs in a pattern that is consistent with the axon behaving mechanically like a piece of “silly putty” that is stretching with a fixed end at the cell body and a pulled end at the growth cone [32]. In addition, forward translocation of microtubules is paired with forward advance of the organelles in the C-domain of the growth cone in *Aplysia* neurons [4,5]. Taken together, these data indicate that docked mitochondria are a reliable and convenient marker for tracking the translocation of the axonal meshwork and microtubules in the growth cone C-domain. 

An important goal in neuronal cell biology is to be able to translate *in vitro* observations to *in vivo* axonal elongation and regeneration [35,36]. In the context of microtubule translocation, there has not yet been a systematic comparison of *in vivo* and *in vitro* observations where substrate and conservation between species have been considered. To determine if *Drosophila* neurons elongate in the same pattern as *Aplysia* and chick sensory neurons [6], we grew them on poly-ornithine and *Drosophila* extracellular matrix proteins (DECM) *in vitro* and monitored the pattern of docked mitochondrial movement. To investigate growth cone mediated axonal elongation *in vivo*, we tracked the movement of docked mitochondria during the elongation of the aCC motor neuron in stage 16 *Drosophila* embryos. We found in all cases, docked mitochondria in the growth cones and along the axon advanced in a pattern consistent with the SAI model. These data suggest that the biophysical mechanism of axonal elongation is widely conserved and occurs by a combination of microtubule assembly and forward translocation of C-domain of the growth cone *in vivo*. 

## Materials and Methods

### Fly stocks

Either *elav* ^*C155*^ *-Gal4*;;*UAS-mitoGFP, dmiro^B682^/TM6B*_*Tb,Sb*_ (a kind gift from Gregory Macleod and Konrad Zinsmaier) [37] or *elav* ^*C155*^ *-Gal4*;;*UAS-mitoGFP* were crossed with *w;;10xUAS-IVS-myr-tdTomato* (Bloomington Stock Collection; Bloomington, IN, USA) to yield *+/elav* ^*C155*^ *-Gal4*;;*UAS-mitoGFP, dmiro^B682^/ 10XUAS-IVS-myr-tdTomato* or *+/elav* ^*C155*^ *-Gal4;;10xUAS-IVS-myr-tdTomato/UAS-mitoGFP* for the *in vivo* imaging experiments. For all other experiments, the w^1118^ line was used as wild-type.

### Preparation of *Drosophila* Extracellular Matrix (DECM)

The *Drosophila* cell line Kc167, acquired from the *Drosophila* Genomics Resource Center, was grown at log phase in HyClone SFX Insect media (Thermo Scientific; Waltham, MA, USA). Note: The *Drosophila* Genomics Resource Center recommends this brand of serum-free media. We found that though the cells grow in serum-free Schneider’s they did so poorly. After 4 d of growth, conditioned media rich in DECM was collected and centrifuged at 500 g for 10 min. Media was decanted and stored at -70°C until further processing. Conditioned media (1.7 L) was processed through Millipore (Billerica, MA, USA) Centricon Plus-70 100kDa Ultracel-PL membrane filter devices at 3000 g down to a final volume of 50 ml (34x concentration) and stored at -70°C. DECM samples were analysed for quantity using the Pierce 660 nm Protein Assay (Thermo Scientific; Waltham, MA, USA) and for quality using SDS-PAGE. Samples were run on a 5-20% polyacrylamide gel at 125 V for 1.5 h and stained with Coomassie Blue. 

### Mass spectroscopy

Prominent bands on the SDS-PAGE gel were subjected to in-gel tryptic digestion. The extracted peptides were then loaded for 5 min onto a Waters Symmetry C18 peptide trap (5 µm, 180 µm x 20 mm) at 4 µL/min in 5% ACN/0.1% formic acid. The bound peptides were then eluted onto a MICHROM Bioresources 0.1 x 150 mm column packed with 3 units 200A Magic C18AQ material over 15 minutes.

### Neuronal cultures

Wild-type *Drosophila* neurons, isolated from embryos of either sex, were used as described [38]. Cells were grown at 25°C and imaged at room temperature in L-15 medium (Life Technologies, Item # 41300039; Grand Island, NY, USA) pH 7.1 supplemented with 0.6% glucose, 1 mM glutamine, 100 U/ml penicillin, 136 µg/ml streptomycin sulfate, 10% fetal calf serum, and N9 growth supplement [27]. Note neuronal outgrowth is more reliable using the powdered version of L-15 noted above, rather than premade liquid L-15. The culture surface (35 mm cell culture dishes, Corning # 430165; Tewksbury, MA, USA) was treated with 0.01% poly-ornithine for 30 min then washed 3x with dH_2_O for 5 min, or with 5 µg/ml DECM for 1 h and rinsed with dH_2_O. Dishes were used immediately following coating.

### Phase imaging

#### Axonal length measurements as a function of DECM concentration

Ten fields of cells of *Drosophila* neurons grown on plastic dishes for 24 h were acquired at each concentration of DECM on the Leica DM IRB using a N Plan L 20x / 0.4 Corr Ph1 ∞ / 0 - 2 / c objective. The length of each neurite longer than the average cell diameter (approximately 10 µm) in the field was measured as the distance between the cell body and tip of the growth cone using ImageJ. 

#### Continuous Measurement of Axonal Elongation

*Drosophila* neurons were plated on plastic dishes coated with either poly-ornithine or DECM and then phase images were captured every 5 min at room temperature for 24 h using either a Leica DM IRB with a N Plan L 20x / 0.4 Corr Ph1 ∞ / 0 - 2 / c objective and an Orca-ER digital camera CCD, model #CA742-95, (Hamamatsu; Hamamatsu, Japan) or a Nikon Diaphot with a Ph2 20x DL 0.4 160 / 0-2 objective and a Spot Diagnostic Instruments RT monochrome Model 2.1.1 camera. In both cases Micro-Manager was used to control the acquisition. Axonal length was measured by tracing the full length of the axon at 30 min intervals in ImageJ. 

### Mitochondria imaging

Mitochondria were labelled in wild-type neurons by adding MitoTracker Red CMX-Ros directly to the culture dish (Invitrogen; Carlsbad, CA, USA) at a final concentration of 5 nM. Cultures were observed with an N Plan L 40x / 0.55 corr Ph2 with an adjustable collar infinity / 0 - 2 / c objective on a Leica DM IRB. Cells were illuminated with a 100 W Xenon lamp attenuated 98% with neutral density filters and visualized with a 49008 ET – mCherry, Texas Red cube (Chroma; Bellows Falls, VT, USA) for MitoTracker. On the Leica DM IRB transmitted light exposure was controlled with a VMM-D3 controller and CS25 shutter (Vincent Associates; Rochester, NY, USA). Fluorescent light exposure was controlled with a Lambda 10-C (Sutter Instruments). Micro-manager software was used to control the shutters and camera (Orca-ER digital camera CCD, model #CA742-95, Hamamatsu; Hamamatsu, Japan). Exposure times were set between 100 to 200 msec. 

### In vivo imaging

Stage 14-15 embryos of either sex were collected from timed egg lays and manually dechorionated. Embryos were oriented at a slight angle with the dorsal surface down on a #1 coverslip lightly coated with embryo glue made by mixing 19:1 chloroform:Spray Mount (3M, St. Paul, MN) and were then lightly coated with 20% chloroform in halocarbon oil 700 (Sigma; St. Louis, MO, USA) to minimize desiccation and muscle contraction. The coverslips were placed directly on a 60x oil immersion objective (NA 1.4) of the Nikon swept field confocal microscope (on a TE2000 platform) and covered with a humidity chamber. After scanning a series of embryos to find one at the correct developmental stage (mid stage 16) and optimal orientation, images were acquired every 2 min at 5% power (set in the NIS software) for the 488 nm line and 100% power for the 561 nm line. Exposure times were 1 s and 20 z-planes with a 0.7 µm step were collected at each time point. ImageJ was used for image analysis as follows. The multiple image planes were Z-projected using the maximum intensity setting at each time point. The limit of the stack was set to exclude the dp1-2 dorsal sensory neurons. In some cases, images were aligned using the Stackreg plugin and axons were straightened using the built-in ImageJ function. To generate kymographs these stacks were resliced and Z-projected using the standard deviation setting. Growth cones advancing faster than 3 µm/h were considered elongating. Mitochondria in the most distal portion of the axons were measured for the rate of advance if they could be tracked for at least 4 frames (i.e. docked for 8 min).

## Results

### *Drosophila* extracellular matrix proteins secreted from the Kc167 cell line promote robust axonal elongation

In order to test whether the SAI model is applicable to a wider range of species and to examine axonal elongation *in vivo*, we turned to the model system *Drosophila* [39]. Because the composition of the substrate has a significant effect on the translocation of long microtubules [30], which is likely to occur through both signalling [40] and differences in adhesiveness [32], we wanted to examine elongation of *Drosophila* neurons *in vitro* on both poly-amines and ECM proteins. While techniques for culturing embryonic *Drosophila* neurons are gradually advancing [38,41], vertebrate laminin does not support the growth of *Drosophila* cells [42,43] and there are currently no commercial sources of *Drosophila* laminin. To acquire *Drosophila* ECM proteins we used the *Drosophila* Kc167 cell line. It secretes the three laminin chains, tiggrin, collagen IV and glutactin [44], and purified laminin isolated from this cell line has been used to culture *Drosophila* cells, neuronal cell lines and neurons [45,46]. Because we were more interested in developing *in vitro* growth conditions that approximated the *in vivo* environment rather than specifically testing how neurons grow on laminin, we characterized the effectiveness of the mixture of DECM proteins produced by Kc167 cells in promoting axonal elongation. 

**Figure 1:**
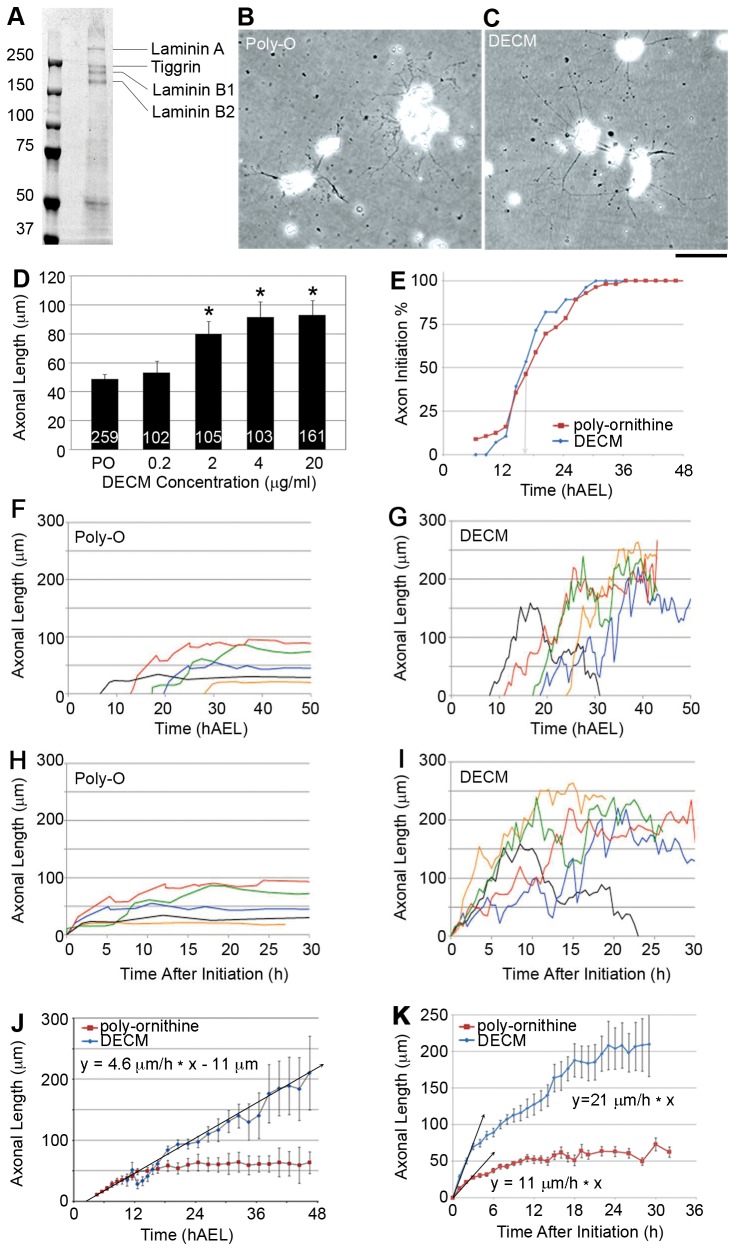
*Drosophila* neurons grow at physiological rates *in* *vitro*. (**A**) Coomassie stain of DECM purified from Kc167 conditioned media. Bands identified as Laminin A, Tiggrin, Laminin B1, and Laminin B2 by mass spectroscopy. Unsequenced band at 50 kDa corresponds to glutactin based on previous reports [44]. Phase images of *Drosophila* neurons grown *in* *vitro* on (**B**) poly-ornithine and (**C**) DECM. Axonal length at 24 hours increases with concentrations of DECM at 2 µg/ml and higher (**D**). The numbers in the bars in (**D**) represent *n* for each group. The graph in (**E**) shows axonal initiation is asynchronous, occurring over a period of ~12 hours, and is not substrate dependent. The arrow marks the time point where 50% of the neurons had initiated axons. Representative examples of growth cone position over time are shown for neurons grown on (**F**) poly-ornithine and (**G**) DECM. By aligning individual growth cone positions so initiation is at t = 0, accurate depictions of cone advance can be more clearly seen. (**H**) poly-ornithine alignment, (**I**) DECM alignment. Averaging axonal length over time without accounting for differences in initiation (**J**) yields rates of elongation similar to previous reports, whereas analysis of synchronized average axonal length (**K**) reveals elongation occurs at rates similar to those observed *in* *vivo*. All error bars are 95% CI. Scale bar = 70 µm.

To verify the composition of the proteins secreted by Kc167 cells, we ran concentrated cell culture supernatant on protein gels and then used mass spectroscopy to indentify the bands with the largest amount of protein (Figure 1A) [44]. To test the effectiveness of DECM in promoting axonal elongation, we collected serum free cell culture supernatant from Kc167 cells, concentrated the total protein to 20 µg/ml, and then compared axonal length of neurons 24 h after plating when cultured on poly-ornithine (Figure 1B) and a series of concentrations of DECM (Figure 1C). We found a concentration of 4 µg/ml DECM causes axonal length to approximately double (91.7 µm +/- 54.3 s.d. n= 103 axons) as compared to poly-ornithine (48.8 µm +/- 26.8 s.d. n= 259 axons), whereas increasing the concentration of DECM to 20 µg/ml did not significantly increase the length of the axons measured at 24 h (Figure 1D; post-hoc Dunnett’s test). Using these numbers to make an estimate of average growth indicates elongation rates of 4 µm/h on poly-ornithine and 7.6 µm/h on 4 µg/ml DECM. The significantly higher rates of growth on DECM are consistent with the well-accepted observation that neurons grow more rapidly on endogenous substrates than poly-amines [47].

### *Drosophila* elongation *in vitro* occurs at rates comparable to rates *in vivo*

While axons of primary embryonic *Drosophila* neurons elongate more rapidly on DECM than poly-ornithine *in vitro*, the rate is slow as compared to the growth of *Drosophila* motoneurons navigating through the periphery *in vivo* [48] (i.e. ~ 20 µm/h). The reason for the slow rate of growth could fall into one of three broad categories. The first is that *Drosophila* neurons *in vitro* are ‘sick’ because key components found *in vivo* are missing in the cell culture media. The second is that while the neurons are healthy, the substrate conditions *in vitro* so poorly match those *in vivo* that rapid rates of elongation are not possible. The third, a more subtle point, is that *Drosophila* neurons do grow rapidly *in vitro*, but this is obscured because of the way growth rates are measured. To address these questions, we continuously monitored fields of neurons for up to 3 days with frames acquired every five min using phase optics to unambiguously track the position of individual growth cones. Our first question was whether a delay in the time of axonal initiation could explain the slow average rates of growth. We considered this as a possibility because in contrast to most systems, the culture of embryonic *Drosophila* neurons involves the plating of neuronal precursors [41,49,50] instead of post-mitotic cells. We found axonal initiation occurred in a 24 hour window after plating, with half of the neurons sprouting axons at 11.5 h after plating (i.e. 16 hours after egg lay (hAEL)) and that substrate had no obvious effect on the average time of initiation (Figure 1E). This suggests that population averages of axonal length will tend to underestimate growth rates because the initiation of axonal elongation in primary embryonic *Drosophila* neurons is asynchronous. 

We then directly assessed the ‘instantaneous’ rate of axonal elongation by tracking the movement of individual growth cones. We found growth cones advanced at a rapid rate following axonal initiation that slowed until axons reached a final stable length. Figures 1F and 1G show representative data for individual neurons grown on poly-ornithine and DECM where growth cone position was monitored for 48 h (n= 68 and 56 axons respectively). Simply averaging the raw data in Figures 1F and 1G produces an average growth graph (Figure 1J) that is very similar to previously reported growth *in vitro* on poly-ornithine [51]. To determine the average instantaneous growth rates, we aligned the time of axonal initiation for each axon as illustrated in Figures 1H and 1I and averaged growth cone position (Figure 1K). For neurons grown on poly-ornithine, elongation initially occurred at 11.1 +/- 1.5 µm/h (ave. +/- 95% c.i., n = 291 measurements of change in growth cone position over 30 min intervals) and then gradually slowed over the next 12 h with length plateauing at 60 µm (Figure 1K). For neurons grown on DECM axonal elongation initially occurred at 20.9 +/- 2.5 µm/h (ave. +/- 95% c.i., n = 736 measurements) and then gradually slowed over the next 30 h with the average length reaching 200 µm (Figure 1K). These data demonstrate that embryonic *Drosophila* neurons *in vitro* elongate at instantaneous rates comparable to *Drosophila* neurons in the periphery *in vivo* [48].

### Anterograde translocation of microtubules during axonal elongation is conserved

**Figure 2:**
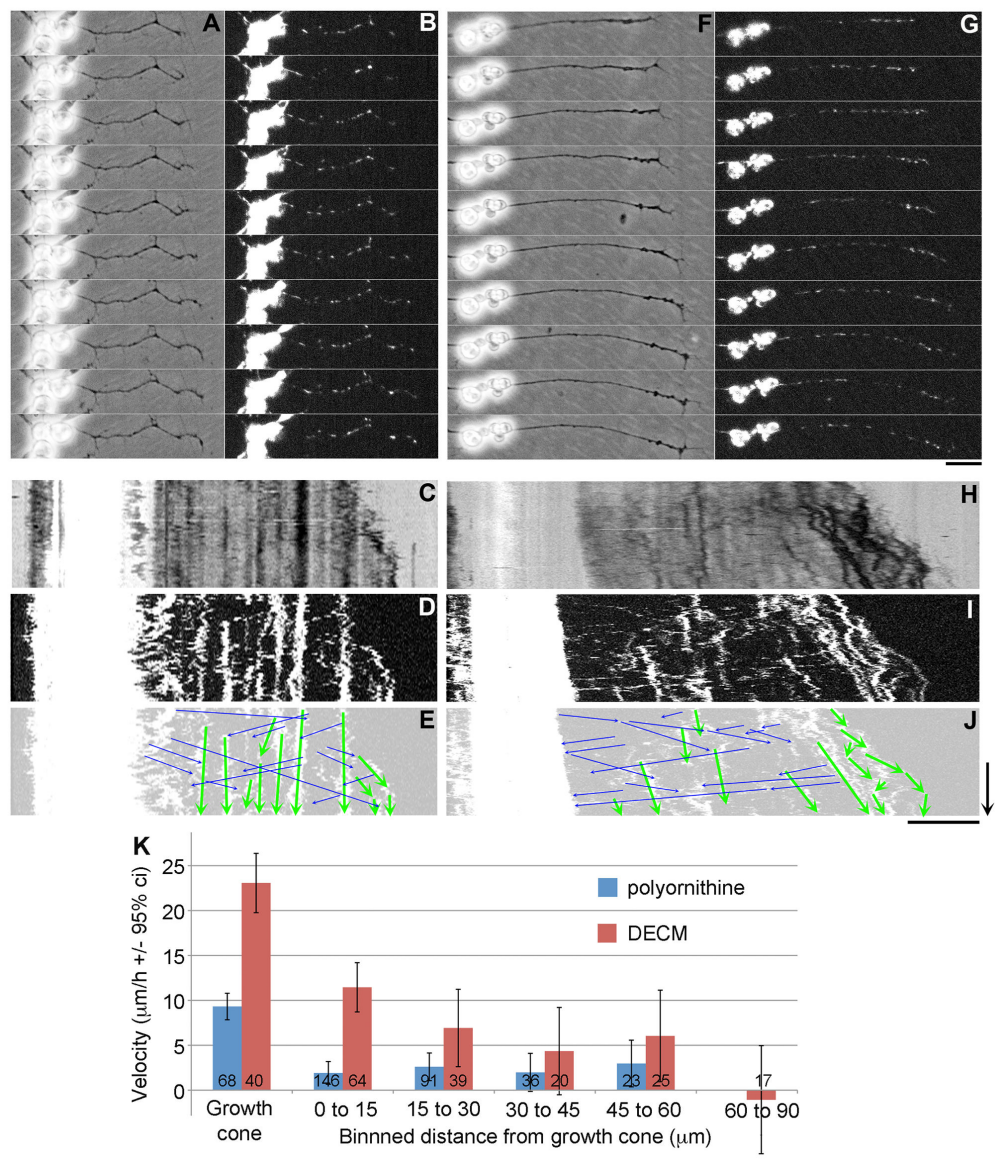
Growth cones advance by forward translocation of the C-domain and axonal framework *in* *vitro*. (**A**) Phase and (**B**) fluorescent images over 1 h of MitoTracker labelled *Drosophila* neurons grown on poly-ornithine. Kymographs of the phase images (**C**) and fluorescent images (**D**) show the position of the growth cone and mitochondria over time. (**E**) Green arrows overlaid on the kymograph illustrate the movement of docked mitochondria and the blue arrows show the tracks of fast transported mitochondria. The corresponding images from a neuron grown on DECM are shown in panels (**F**-**J**). Time arrow = 30 min and scale bar is 10 µm for both the time-lapse images and kymographs. (**K**) Quantitative analysis of the velocity of docked mitochondria plotted against distance from the growth cone. Errors bars are 95% confidence intervals. The numbers at the base of the bars denote the number of mitochondria analyzed in each bin. The growth cone is defined as the first 5 µm of axon.

As a means to assess if *Drosophila* neurons elongate by microtubule assembly at the tip of a stationary array of microtubules or by combination of microtubule assembly and translocation as is seen in other species [6] we monitored the movement of docked mitochondria in neurons plated on poly-ornithine and DECM at 1 min intervals for 1 to 2 hours. As the transport velocity of kinesin and dynein occurs at a characteristic rate of ~ 0.1 - 1 µm/s (i.e. 360 - 3600 µm/h) whereas axonal elongation and stretching occurs at 1-50 µm/h, distinguishing between fast transported and slowly moving docked mitochondria is straightforward [3]. Examples of what we defined as either docked or fast transported mitochondria are shown as green and blue arrows, respectively, in the mitochondrial kymographs (Figures 2E and 2J). On poly-ornithine (18 neurons analyzed), we found docked mitochondria along the axon moved at a rate of 2 to 3 µm/h (Figure 2B, D, E, and K). In contrast, on DECM (40 neurons analyzed), mitochondria along the length of the axon moved at 5 - 10 µm/h in a velocity gradient that was highest at the growth cone (Figure 2G, 2I, J, and K) (Movie S1). On both poly-ornithine and DECM, we observed that mitochondria in the growth cone advanced with the growth cone, though at a higher rate on DECM. Therefore, similarly to what is found in *Xenopus* neurons on laminin [30], DECM increases translocation of the axonal cytoskeletal meshwork. Together this suggests that substrate effects on neuronal outgrowth are conserved [6].

### Growth cones advance by anterograde translocation of the axonal meshwork *in vivo*

**Figure 3:**
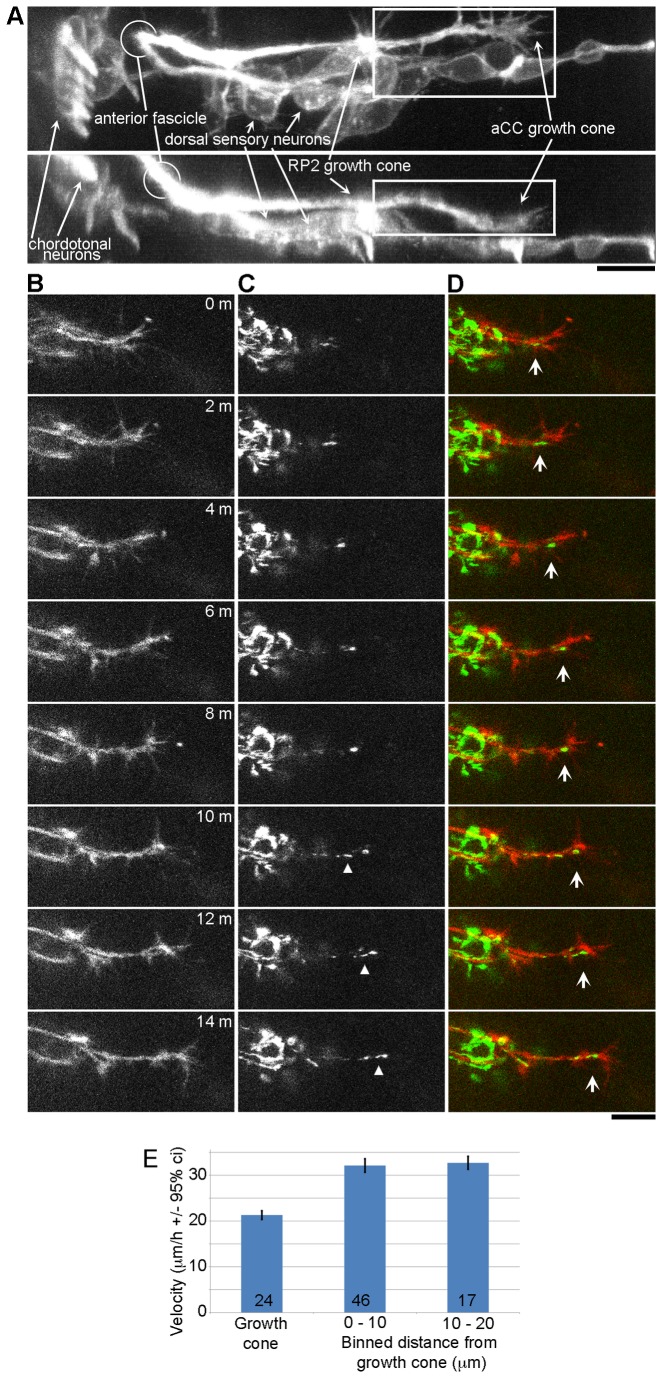
Growth cones advance by forward translocation of the C-domain and axonal framework *in* *vivo*. (**A**) A 3D reconstruction of late stage 16 embryo expressing the membrane marker myr-tdTomato in the nervous system via *elav-Gal4*. After the intersegmental axon of the aCC neuron passes the point where the RP2 axon forms a synapse on muscle 2, it is in a region free of other axons and the cell bodies of surrounding sensory neurons. The box indicates the region of the aCC motor axon that was used for 3D analysis of mitochondrion advance. (**B** - **D**) Time-lapse series of an elongating *Drosophila* aCC motor neuron in stage 16 embryo of the genotype +/elav-Gal4;;*UAS-mtGFP*, *dmiro* ^B682^/ *IVS-10XUAS-myr-tdTom*, shown at 2 min intervals. (**B**) myr-tdTomato (red in **D**) labels neuronal plasma membranes. (**C**) mitoGFP (green in **D**) labels mitochondria. The arrow shows a mitochondrion in growth cone. In the last half of the series a mitochondrion docks in the distal axon (triangle in **B**) and advances. (**E**) Average velocity of docked mitochondria in the growth cone, defined as the last five µm of the axon, and in binned regions along the distal axon. Because the RP2 axon is fasciculated with the aCC axon (**A**), only mitochondria in the last 25 µm of the aCC axon were analyzed. Error bars show the 95% confidence intervals. The number at the base of the bar is the number of docked mitochondria that were analyzed. Scale bars = 10 µm.

We next tested whether microtubule translocation in the growth cone and distal portion of the axon occurs similarly *in vivo* by monitoring docked mitochondrial movement in the aCC pioneer neuron in stage 16 *Drosophila* embryos [52]. This neuron [53] originates in the CNS in stage 10 embryos, approximately 10 hours after egg lay. The elongation of the aCC axon occurs over a time and distance of approximately 6 hours and 200 µm [54] over a basal lamina consisting of roughly a dozen proteins secreted by the fat body and hemocytes including laminin, tiggrin, glutactin and perlecan [55]. The focus of our studies was in the region of muscles 1 and 2, past the synaptic termination point of the RP2 motor neuron. We chose to follow the aCC growth cone in this region because growth occurs along a plane close to the body wall through a region in the embryo that allows visual isolation of the growth cone (Figure 3A, Movie S2). 

To track axonal elongation and the movement of docked mitochondria in the distal axon and growth cone, we co-expressed the plasma membrane marker myr-tdTomato and mitochondrially targeted GFP [33] using the pan-neuronal Gal4 driver *elav*. Docked mitochondria were defined as those that maintained their relative position along the axon for at least 8 minutes and moved at a velocity of less than 100 µm/h. In our initial observations using +/elav-Gal4;;*UAS-mitoGFP*/*IVS-10XUAS-myr-tdTom* embryos, we observed only 1 - 2 docked mitochondria per axon. We therefore sought a genetic means to increase the number of docked mitochondria*. dmiro* ^*B682*^ mutants have reduced fast mitochondrial transport [37], which we reasoned would increase the frequency of mitochondria docking to the axonal meshwork. We used heterozygous *dmiro* ^B682^ embryos, which increased the number of docked mitochondria to 2-3 per axon (Table 1). In total we imaged 35 growth cones (21 with the genotype +/elav-Gal4;;*UAS-mitoGFP*/*IVS-10XUAS-myr-tdTom* and 14 with the genotype +/elav-Gal4;;*UAS-mitoGFP, dmiro* ^B682^/*IVS-10XUAS-myr-tdTom*). We found no differences in the rates of growth cone advance or docked mitochondrial movement between *dmiro* ^*wt*^ and heterozygous *dmiro* ^*B682*^ so the data were pooled (Table 1). The pooled average rate of growth cone advance was 20.0 +/- 3.0 µm/h (ave +/- 95% c.i., n = 35). Likewise the movement of docked mitochondria in the growth cone, defined as the distal most 5 µm of the axon, had the same average rate of advance (Figure 3E). Along the next 20 µm of axon, docked mitochondria advanced at an average rate of ~30 µm/h. The higher rate of docked mitochondrial movement, as compared to the rate of growth cone advance (Figure 3E), appears to occur because translocation of docked mitochondria continues when growth cones briefly pause [3,56]. For example, the triangle in Figure 3C points out a docked mitochondrion that is advancing more rapidly than the growth cone. In all instances where a mitochondrion was found in the growth cone it advanced simultaneously with the growth cone (arrow, Figure 3B-D; Movie S3). These data indicate that growth cones of *Drosophila* motor neurons advance by forward translocation of the axonal cytoskeletal meshwork and organelle rich C-domain. 

**Table 1:** Rates of growth cone and docked mitochondrial advance are the same in *dmiro* ^wt^ and heterozygous *dmiro* ^B682^ axons *in vivo*.

Genotype	**GC rate (µm/h)**	**Mito rate (µm/h)**	**Mito per axon**
**dmiro^+/+^**	20.5 +/- 3.7 (21)	29.0 +/- 7.5 (32)	1.6 +/- 0.6 (21)
***dmiro^+/-^***	19.3 +/- 5.9 (14)	31.0 +/- 8.1 (38)	2.4 +/- 0.6 (14)
**combined**	20.0 +/- 3.0 (35)	30.1 +/- 5.4 (70)	

All values reported as ave +/- 95% CI. Values in parenthesis represent n values. No significant differences were found between growth cone or mitochondria rates of advance (P = 0.707 and 0.732, respectively, by unpaired two tail, t-test). The number of docked mitochondria in the distal 30 µm of the axon was significantly higher in the heterozygous dmiroB682 axons (P = 0.05).

## Discussion

By monitoring the movement patterns of docked mitochondria to track the subcellular movement of the axonal meshwork during axonal elongation, our data suggest that the influence of substrate on microtubule translocation during axonal elongation is shared between species and that the forward translocation of microtubules in the axon contributes to the advance of the C-domain and hence axon elongation, both in culture and *in vivo*.

### Extracellular matrix proteins from the Kc167 cell line provide useful culture substrates to study neuronal processes

There has been a surge of interest in the development of *in vitro* neuronal culture techniques in *Drosophila* [38,57–60]. This provides new avenues to combine well established molecular /genetic tools with timelapse microscopy [3], super-resolution microscopy [61], ultrastructural analysis [62], *in vitro* RNAi [63], and biophysical approaches [6]. In terms of developing *in vitro* culture systems that allow the exploration of the wider range of parameters known to be present *in vivo*, the inclusion of physiologically relevant ECM proteins is important [55]. Our work here demonstrates a straightforward means to concentrate and apply DECM in tissue culture and describes the concentration range over which axonal elongation is promoted. We also note DECM can be stored at -70°C for at least a year, which is both convenient and decreases experimental variability; two advantages that are important for both small and high throughput gene disruption experiments. While supernatant collected from Kc167 cells is a convenient source of *Drosophila* extracellular matrix proteins, it contains a complex mixture of proteins [44]. While we view this as an advantage in our studies, in the context of understanding the process of axonal elongation, it will be important to systematically analyze the function of the individual ECM proteins and their receptors to assess their roles in mediating adhesion [15] and their modulation of signaling pathways [55]. 

### *Drosophila* neurons elongate robustly but briefly *in vitro*

Based on our experience with chick and rat neurons [27,64], we were initially struck by the slow growth of *Drosophila* neurons *in vitro*. We found (Figure 1J), as others have reported [50,51,57], an average rate of growth of ~ 3 - 5 µm/h. In contrast, *Drosophila* growth cones advance at a rate of 20-30 µm/h *in vivo* [48] (Figure 3E). By unambiguously tracking individual growth cones and accounting for asynchronous axon initiation in culture (Figure 1E), we found instantaneous growth rates of ~10 µm/h on poly-ornithine and ~20 µm/h on DECM (Figure 1K), the latter of which is within the window of growth rates observed *in vivo*. DECM will thus be an important tool in future *in vitro* studies to achieve the higher velocities observed *in vivo.*

While we found *Drosophila* neurons grow rapidly *in vitro*, for individual neurons this occurred for a time period of less than 24 h (Figure 1K). While it is well accepted that as neurons mature they lose their capacity for elongation and regeneration, what controls the intrinsic decrease in growth potential is poorly understood. Two of several possibilities are that neurons have a means to measure axonal length [65] and switch off growth when a set distance has been reached. In addition, there may be an internal clock that acts independently of axonal length and activates maturation after a set time. While we have previously suggested that a length sensor controls axonal transport in *Drosophila* larvae [66], our data here suggest a clock, similar to that which controls differentiation, electrophysiological properties, and neuronal process morphologies [67], may regulate the transition to maturity for *Drosophila* neurons *in vitro* [50]. In support of this we note that if a length sensor solely regulated the cessation of elongation, neurons grown on poly-ornithine would be predicted to sustain elongation for a longer time than neurons grown on DECM (Figure 1K). *Drosophila* provides an excellent platform for studying changes in gene and protein expression and because their neurons develop rapidly, this system has the potential to be useful for studying why neurons lose their capacity for growth over time. 

### The pattern of axonal elongation is similar between *Drosophila* and other species *in vitro*

As a prerequisite to analyzing the pattern of axonal elongation *in vivo*, we felt that it was important to establish that *Drosophila* neurons grow in a manner similar to other types of neurons *in vitro*. If they did it would suggest that regardless of the results we observed *in vivo*, they would applicable to other species. Closely related to this question was the issue of whether *in vitro* axonal elongation recapitulates growth *in vivo*. While this is an unspoken assumption, it has not been systematically validated in terms of whether microtubules are stationary or translocate forward during axonal elongation. Two important aspects of this problem are that the rate of microtubule translocation varies along the length of the axon and the adhesiveness of the substrate modulates translocation velocity [32]. Thus to characterize microtubule translocation in *Drosophila* neurons, examination of one point along the axon on one type of substrate is not sufficient. To address these issues we grew *Drosophila* neurons on poly-ornithine and DECM *in vitro* and monitored the pattern of docked mitochondrial movement along the length of the axon (Figure 2K). On both substrates, we observed that the rate of forward translocation was higher in the growth cone than along the length of the axon. In addition, the overall velocity was higher in neurons grown on DECM than on poly-ornithine. This movement pattern and response to growth on ECM protein have both been observed in chick sensory [32] and *Xenopus* neurons [30,56]. Together these observations indicate that the pattern physical mechanism underlying microtubule translocation [6] *in vitro* is similar between *Drosophila* and other species. 

### Growth cones advance by forward translocation of the axonal meshwork *in vivo*

While our analysis of mitochondrial movement (Figure 2K) confirms that *Drosophila* neurons, like chick [3], rat [64], and *Aplysia* neurons [4,5], elongate by forward translocation of microtubules, these experiments were all carried out *in vitro*. *In vivo* analysis of microtubule translocation in Zebrafish and grasshopper Ti1 pioneer neurons, in contrast revealed microtubules are stationary along the axon [68,69]. One possibility that can explain these differing results is that microtubule translocation only occurs *in vitro* and because axonal elongation is a highly conserved process this is an ‘artifact’ that is seen in various species. To investigate we tracked the movement of docked mitochondria in the growth cone and distal axon in *Drosophila* embryos *in vivo* (Figure 3). We found mitochondria advanced in a pattern consistent with anterograde translocation of the axonal meshwork, but in turn this raises the question of why the *in vivo* data conflict. We suggest the underlying reason is that we examined translocation near the growth cone (Figure 3), whereas the previous studies [68,69] focused on the region of the axon closer to the cell body to test a now defunct theory about slow axonal transport called the Structural Hypothesis [70–72]. Previous *in vitro* studies in chick [3] and *Xenopus* neurons [30,56], as well as our *in vitro* studies here (Figure 2K), all show that the cytoskeletal meshwork moves more slowly or is stationary close to the cell body, but moves forward near the growth cone [6]. Biophysical analysis suggests this occurs because axons stretch and forces that move the axonal meshwork forward are dissipated along the axon through adhesions [32]. Thus we see no conflict between our *in vivo* observations and prior studies in Zebrafish and grasshopper [68,69]. While the similarity between the pattern of elongation we observe *in vitro* (Figure 2K) between *Drosophila* and chick neurons [3] suggests our *in vivo* findings may be relevant to other species, because of the complexity of axonal elongation *in vivo* it will be important to explicitly examine growth cone motility in other systems (e.g. Zebrafish, grasshopper, chick, mouse) and cell-types. 

### Toward a comprehensive model of axonal elongation

While microtubule assembly is critical for axonal elongation [26,73,74], the contribution of microtubule translocation has only recently become appreciated [6]. Moving beyond the debates of whether long microtubules are stationary or move, understanding the mechanisms that underlie their translocation is the next major question. In the context of the findings noted above, we propose two highly speculative models that can account for microtubule translocation. In both, forces generated by molecular motors such as myosin [75], dynein [76], mitotic kinesins [24], and Kinesin-1 [77], not only move short microtubules by stop-and-go transport [24], but also drive the slow advance of the long microtubule array [6,78]. In the first, these motors generate a net force that pushes microtubules along the axon forward and myosin II driven actin retrograde flow in the growth cone acts as a dynamic barrier that blocks their advance [79–83]. Part of the appeal of this model is that it has been known for decades that axons can elongate when actin is disrupted [84]. In addition, recent experiments in *Drosophila* have revealed that Kinesin-1 is capable of sliding microtubules out of the neuronal body during the process of neurite initiation [77]. Nonetheless, this model of axonal elongation seems incomplete. When the actin cytoskeleton is intact, detachment of growth cones from the substrate [85] or axonal severing [86] leads to axonal retraction driven by actomyosin contractile forces generated along the axon [87,88]. Furthermore, it is well accepted that when actin is intact growth cones pull [89] the substrate rearwards while pulling the C-domain forward [6]. To explain these observations we suggest that while microtubules along the axon push forward, contractile forces generated along the axon are larger [87,88] and thus retraction of the axon occurs when the growth cone is detached from the substrate [85]. In the growth cone, coupling between actin and microtubules [2] sweeps microtubules that polymerize or translocate into the P-domain back [90–92], yet the net force generated by the growth cone pulls microtubules in the C-domain [4,5] and along the axon forward [3]. The key difference between the two models is that in the first the net force generated along the axon by the combined actions of the microtubule and actin cytoskeleton pushes forward and forces generated in the growth cone restrain this advance. In the second, the net forces generated along the axon pull the growth cone rearwards, while the growth cone pulls forwards. In summary, our work suggests models of growth cone motility need to incorporate microtubule translocation in addition to assembly, raises the question of what powers translocation, and provides tools for testing various models. 

## Supporting Information

Movie S1 **Mitochondrial movement in *Drosophila* neurons *invitro*.** Phase and fluorescent images were acquired of MitoTracker labeled neurons grown on poly-ornithine and DECM at 1 min intervals for 1 h.
(MOV)

Movie S2 **Rotation of a three-dimensional reconstruction of the aCC motor neuron and surroundings at late stage 16.** Expression of the membrane marker myr-tdTom driven by the *elav*-Gal4 promoter shows sensory and motor neurons in the region of muscles 3, 2 and 1. The aCC motor neuron traverses the anterior fascicle along with the RP2 neuron. Both continue in parallel past the chordotonal neurons and a cluster of dorsal sensory neurons. The RP2 neuron forms a synapse with muscle 2 just above the dorsal sensory neurons while the aCC neuron continues growing for approximately 30 more µm until it synapses with muscle 1. This final stage of elongation of a singular growth cone was used for live *in* *vivo* imaging. Scale bar = 20 µm.
(MOV)

Movie S3 **Mitochondria advance with the growth cone *invivo*.** A time-lapse movie at 2 min intervals showing elongation of the aCC motor neuron in a *Drosophila* embryo during stage 16 of development. The neuronal membranes are labeled red with myr-tdTomato and the mitochondria are labeled green with mitoGFP.
(MOV)
